# Controlling Macrophage Polarization to Modulate Inflammatory Cues Using Immune-Switch Nanoparticles

**DOI:** 10.3390/ijms232315125

**Published:** 2022-12-01

**Authors:** Ana F. Almeida, Margarida S. Miranda, Adriana Vinhas, Ana I. Gonçalves, Manuela E. Gomes, Márcia T. Rodrigues

**Affiliations:** 1 3B’s Research Group, I3Bs–Research Institute on Biomaterials, Biodegradables and Biomimetics, University of Minho, Headquarters of the European Institute of Excellence on Tissue Engineering and Regenerative Medicine, AvePark, Parque de Ciência e Tecnologia, Zona Industrial da Gandra, 4805-017 Barco, Guimarães, Portugal; 2 ICVS/3B’s–PT Government Associate Laboratory, 4710-057 Braga/Guimarães, Portugal

**Keywords:** inflammation, cytokines, SPION, macrophages, targeted delivery, magnetically assisted technologies

## Abstract

The persistence of inflammatory mediators in tissue niches significantly impacts regenerative outcomes and contributes to chronic diseases. Interleukin-4 (IL4) boosts pro-healing phenotypes in macrophages (Mφ) and triggers the activation of signal transducer and activator of transcription 6 (STAT6). Since the IL4/STAT6 pathway reduces Mφ responsiveness to inflammation in a targeted and precise manner, IL4 delivery offers personalized possibilities to overcome inflammatory events. Despite its therapeutic potential, the limited success of IL4-targeted delivery is hampered by inefficient vehicles. Magnetically assisted technologies offer precise and tunable nanodevices for the delivery of cytokines by combining contactless modulation, high tissue penetration, imaging features, and low interference with the biological environment. Although superparamagnetic iron oxide nanoparticles (SPION) have shown clinical applicability in imaging, SPION-based approaches have rarely been explored for targeted delivery and cell programming. Herein, we hypothesized that SPION-based carriers assist in efficient IL4 delivery to Mφ, favoring a pro-regenerative phenotype (M2φ). Our results confirmed the efficiency of SPION-IL4 and Mφ responsiveness to SPION-IL4 with evidence of STAT6-mediated polarization. SPION-IL4-treated Mφ showed increased expression of M2φ associated-mediators (IL10, ARG1, CCL2, IL1Ra) when compared to the well-established soluble IL4. The ability of SPION-IL4 to direct Mφ polarization using sophisticated magnetic nanotools is valuable for resolving inflammation and assisting innovative strategies for chronic inflammatory conditions.

## 1. Introduction

Inflammation is a dynamic process triggered by the immune system in response to injury or infection. However, the uncontrolled activation of immune cells results in persistent inflammatory signals, with deleterious consequences for tissue regeneration. Resolving inflammation is thus a challenge to be overcome with impact in the management of chronic inflammatory diseases [1], autoimmune processes [2], and neurodegenerative disorders [3]. A controlled and timely inflammatory response is mediated by macrophages (Mφ), which respond to environmental cues by acquiring specialized functional phenotypes [4]. The activation of macrophages into pro-inflammatory (M1) or anti-inflammatory (M2) phenotypes has an impact on the cascade of inflammatory events with a direct influence on regenerative outcomes. Targeting macrophage polarization constitutes a disruptive approach for the treatment of inflammation-related conditions. These pathologies are often treated with anti-inflammatory drugs or directed against pro-inflammatory cytokines that suppress the inflammatory response without disrupting the pathological process. Thus, strategies to modulate inflammation fostering resolution anticipate attractive prospects for the management of chronic diseases with significant health and socioeconomic impacts.

Interleukin-4 (IL4) is a key regulator in humoral and adaptive immunity and a well-known promoter of alternatively activated M2 macrophages (M2φ) [5]. The presence of IL4 in tissue niches suppresses tumor necrosis factor-alpha (TNFα), interleukin-1 (IL1), and prostaglandin E2 (PGE2) levels after an inflammatory stimulus [6]. Unlike M1 macrophages (M1φ) which produce pro-inflammatory mediators and contribute to inflammation, M2φ drives anti-inflammatory and pro-repair mechanisms [5]. IL4-mediated actions depend on the receptor alpha chain IL4Rα1 [7] and on the signal transducer and activator of transcription 6 (STAT6) [8]. The activation of STAT6 by phosphorylation leads to its binding to DNA, which regulates the transcription of anti-inflammatory molecules such as interleukin-10 (IL10) and arginase-1 (ARG1). Furthermore, IL4/STAT6 signaling dampens macrophage responsiveness to inflammatory stimuli [9], which could elicit a targeted and precise in situ response to prevent chronic inflammatory niches and stimulate healing. The potential therapeutic value of IL4 has been widely investigated through the local administration of IL4 in arthritis [10,11] and for chronic inflammation [12]. Nevertheless, poor targeting of bioactive molecules to the cells of interest, diminished bioactivity, lower efficacy due to instability, and short half-life in biological fluids are obstacles to overcome. Therefore, an efficient delivery system is required to precisely immunoregulate Mφ function at the injury site by self-limiting inflammatory signals, without impairing healing outcomes.

Magnetically assisted technologies [13,14,15] offer highly sensitive and multimodal tools that permit the finely tuned delivery of cytokines with cell-targeted action. External magnetic fields (EMFs) exhibit excellent tissue penetration and low interference with the cellular environment due to the not inherent magnetic nature of cells and tissues. Moreover, the contactless nature of EMF action minimizes the possible harmful effects on cell integrity and viability. EMFs can be generated from user-friendly and inexpensive instrumentation (e.g., permanent magnets) and are poorly influenced by features as ionic strength, surface charges, pH, and temperature, offering compelling arguments for magnetic targeting and precision therapeutic nanoplatforms in human-driven applications. 

Although IL4 is a well-established M2φ switch, there is a lack of research on IL4 delivery using particulate carriers. Such systems would enable cell and receptor magnetic targeting strategies for the in situ modulation of the macrophage phenotype to locally discourage abnormal inflammatory signals and reestablish pro-healing environments. Therefore, we hypothesized that superparamagnetic iron oxide nanoparticles (SPION) decorated with IL4 (SPION-IL4) favor M2φ via magnetically guided IL4 presentation. By targeting Mφ, which has a coordinated action over inflammatory cascades, SPION-IL4 could assist in the contactless control of inflammatory cues in injured or pathological environments. Here, SPION-IL4 were fabricated and investigated downstream of the IL4 receptor in Mφ. The activation of STAT6 and the gene and protein inflammatory mediators were assessed with different concentrations of SPION-IL4 and compared with carrier-free IL4 (Exo IL4) exogenously supplemented to the culture medium. We foresee improved and more effective SPION-mediated therapeutics in precision Mφ targeting for the immunomodulation of persistent inflammatory environments.

## 2. Results and Discussion

### 2.1. Production and Characterization of IL4-Functionalized SPION

**Figure 1:**
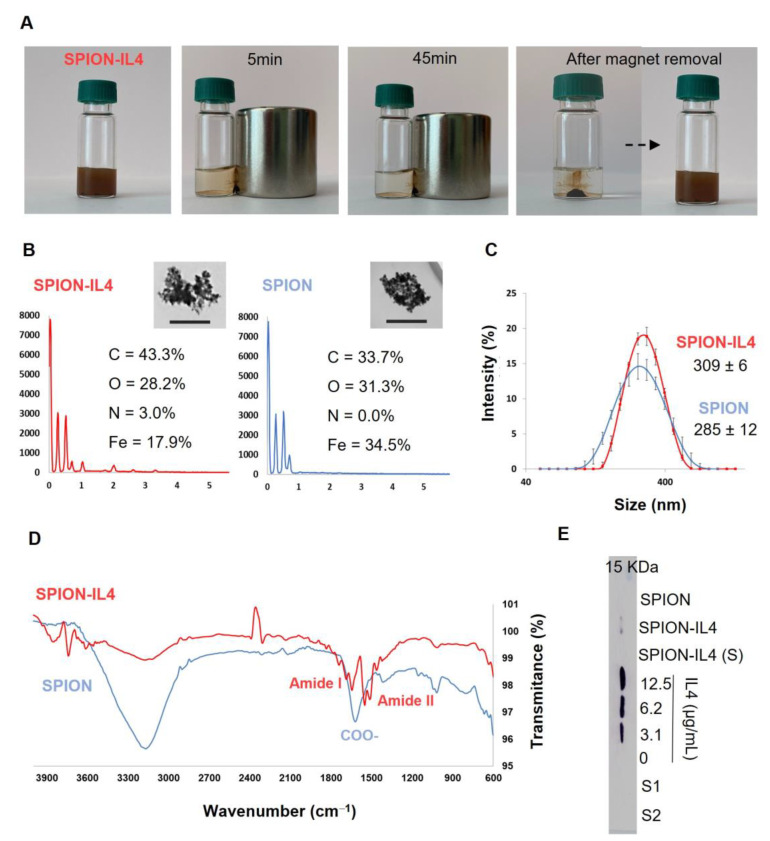
Physicochemical characterization and evaluation of the functionalization efficiency of SPION-IL4. (**A**) Representation of the magnetic responsiveness of SPION-IL4. The magnetite iron oxide core of SPION-IL4 gives a brown color to the solution, and when the solution is placed in an EMF generated by a permanent neodymium magnet (500 mT) positioned on the right side of the flask, SPION-IL4 develops a strong magnetization that persists over time. Owing to the superparamagnetic properties of SPION, after magnet removal, SPION lose their magnetic memory (lack of net magnetization), allowing SPION to significantly avoid magnetic aggregation, which is advantageous for their use in biomedical applications; (**B**) EDS spectra of SPION-IL4 (red) and non-functionalized SPION (blue) and respective scanning transmission electron microscopy (STEM) images, scale bar = 200 nm; (**C**) Particle hydrodynamic size distribution; (**D**) Full range FTIR spectrum highlighting the bands replacement at the IL4 fingerprint region; (**E**) Western blotting for the detection and quantification of IL4. The IL4 band represents soluble/unbounded IL4, while the absence of an IL4 band indicates SPION-bonded IL4 in SPION-IL4 and supernatants S1 and S2. SPION-IL4 (S) served as a control for SPION-IL4 binding. IL4 standard solutions (0 to 12.5 µg/mL) were used for the detection of soluble IL4. S1 represents the supernatant from the IL4 incubation solution with SPION whereas S2 represents the supernatant from the first purification step. IL4 quantification was performed using Image J 1.52o software for band quantification (Supplementary Materials).

Functionalization of SPION with IL4 was conducted using 1-ethyl-3-(3-dimethylaminopropyl) carbodiimide/N-hydroxysuccinimide (EDC/NHS) chemistry. The magnetic response of SPION-IL4 to EMF was evaluated, and SPION-IL4 exhibited magnetic responsiveness (Figure 1A). The inorganic and organic components of the non-functionalized SPION were evaluated using thermal gravimetric analysis (TGA). The mass percentage of the inorganic component was 78 ± 9% *w*/*w* and that of the organic component was 22 ± 9% *w*/*w*, confirming the presence of dextran-COOH in the shell of the SPION (Supplementary Materials, Figure S1).

A stable amide (-CONH-) covalent linkage was selected for the magnetic presentation of IL4 to macrophages. This conjugation method has been used for the functionalization of nanomaterials with proteins, peptides, antibodies, and nucleic acids [16,17,18,19] offering advantages such as a high coupling efficiency and nanoparticle stability. Energy-dispersive X-ray spectroscopy (EDS) analysis of SPION-IL4 showed the presence of nitrogen (N), an indication of the IL4 protein, which was absent in the SPION condition (no IL4 functionalization) (Figure 1B). Moreover, SPION-IL4 was found to contain a higher mass percentage of carbon (C), whereas that of oxygen (O) was lower than that of SPION. These results are in agreement with published reports, where a titanium implant surface coated with poly(dopamine), in which IL4 was immobilized, the levels of N and C were higher in the presence of IL4 in contrast with the O levels [20]. Dynamic light scattering (DLS) analysis showed that there was a slight increase in the hydrodynamic diameter of SPION-IL4 (309 ± 6 nm) in comparison with SPION (285 ± 12 nm) (Figure 1C), which could be attributed to the presence of IL4 in SPION-IL4. Additionally, zeta potential analysis showed that SPION-IL4 has a more positive surface charge (−18 ± 1 mV) than SPION (−23.3 ± 0.9 mV) owing to the linkage to IL4, which has an isoelectric point of 8.3 and thus is a slightly positively charged protein in aqueous media. Both SPION-IL4 and SPION showed a monodisperse size distribution with a polydispersity index (PDI) <0.21.

The functionalization of SPION with IL4 was further confirmed by Fourier-transform infrared (FTIR) spectroscopy (Figure 1D). The FTIR analysis for SPION showed a band at ~1620 cm*^−^*^1^, which is due to the stretching vibrations of the carbonyl bond of the carboxylic acid group (COOH) in dextran. When comparing the SPION to the SPION-IL4 spectrum, this band was replaced by two bands at ~1650 cm*^−^*^1^ and ~1550 cm*^−^*^1^. The band at ~1650 cm*^−^*^1^ is due to carbonyl bond stretching vibrations, whereas the band at 1550 cm*^−^*^1^ represents N–H bond bending vibrations. Thus, the substitution of a COOH representative band in SPION by two amide bond representative bands indicates the successful functionalization of SPION with IL4. The absence of a 15 kDa band associated with IL4 molecular weight in the sodium dodecyl sulfate–polyacrylamide gel electrophoresis (SDS-PAGE) (Supplementary Materials, Figure S2) in the SPION-IL4 as well as in the supernatants (supernatant from the IL4 incubation solution with SPION (S1) and supernatant from the first purification step (S2)) reinforces the high binding efficiency of IL4 to SPION and the high purity of the IL4 functionalized SPION. Western blotting results also confirmed that IL4 was efficiently bound to SPION (Figure 1E). Despite the presence of a mild IL4 band in SPION-IL4, likely due to electrical charge degradation of SPION-IL4 during electrophoresis separation [21], the absence of an IL4 band in S1, S2, and SPION-IL4 (S) supports the binding of IL4 to SPION in SPION-IL4. Moreover, the IL4 standard curve (0–12.5 µg/mL) also enabled the calculation of the concentration of soluble IL4 in the SPION-IL4 samples as being 0.25 µg/mL, thus estimating the efficiency of functionalization of 97.5% in SPION-IL4 particles (Supplementary Materials, Figure S3).

### 2.2. In Vitro Cytotoxicity Assessment of SPION-IL4

**Figure 2:**
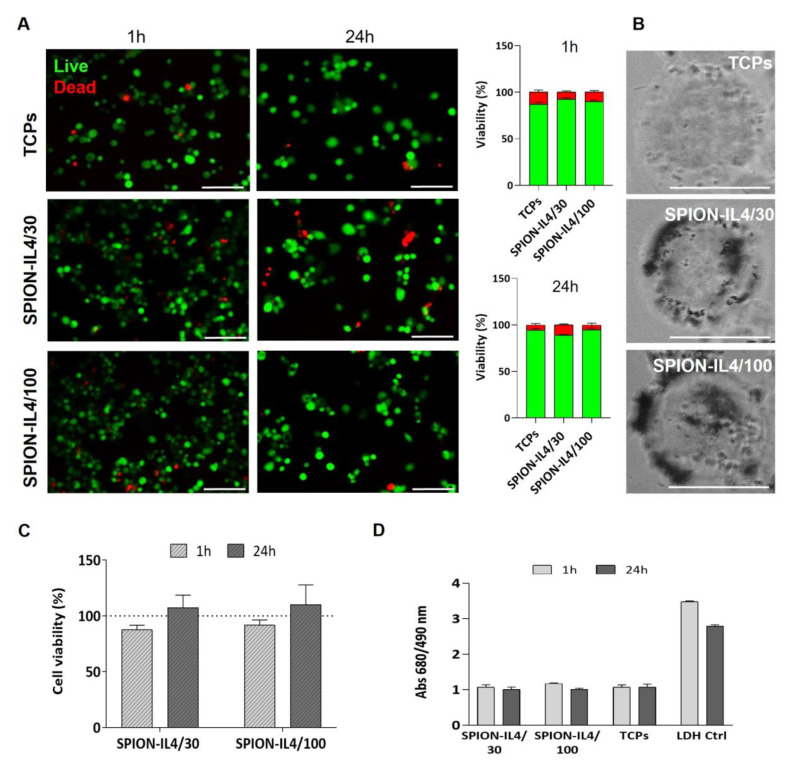
Viability and cytotoxicity assessment of THP1-derived macrophages treated with SPION-IL4 at 30 µg/mL (SPION-IL4/30) or 100 µg/mL (SPION-IL4/100) at two time-points. (**A**) Representative images and quantification of Calcein AM-labeled (live, green) and Propidium iodide-labeled (dead, red) cells. Scale bar = 50 µm. The average number of live/dead cells per field was analyzed and is represented in the graphs; (**B**) Brightfield images of THP1 treated for 24 h with SPION-IL4. The SPION-IL4 on the cells are identified by the dark regions, which are not observed in the SPION-IL4-free cells (TCPs). Scale bar = 25 µm; (**C**) MTS assay and (**D**) LDH release profile. Graph bars are represented as mean ± SE. All conditions were EMF stimulated for 1 h or 24 h using a magnefect nano device (350 mT/well).

SPION have been investigated concerning their biocompatibility, non-toxicity [22,23], and non-immunogenicity properties as well as their biodegradability and clearance in vivo [23,24]. SPION were shown to be safe, without causing deoxyribonucleic acid (DNA) damage with concentrations up to 40 µg/cm^2^ [25] and lactate dehydrogenase (LDH) leakage assay did not produce cytotoxicity up to 100 µg/mL [26]. In the present study, different concentrations of SPION-IL4 (30 µg/mL and 100 µg/mL of iron referent to SPION-IL4/30 and SPION-IL4/100, respectively) were investigated to confirm SPION-IL4 non-toxic behavior towards THP1-derived macrophages. Independently of the SPION-IL4 concentration and the time-point studied (1 h and 24 h), no significant differences (*p* > 0.05) were observed in cellular viability (Figure 2A) despite the close interaction with the cells after 24 h (Figure 2B). The live/dead results were consistent with those of the MTS assay (Figure 2C), and LDH leakage assay (Figure 2D).

### 2.3. SPION-IL4 Incentivizes the Phosphorylation of STAT6 and Influences the Synthesis and Expression of IL4 and IL4Rα

From the physico-chemical characterization performed, we estimated an IL4 loading of 9.7 µg/mL in SPION-IL4/30 and SPION-IL4/100. We also determined the availability of IL4 per cell under all conditions for comparison of the efficiency of SPION-IL4 and soluble IL4 (Exo IL4) administration to Mφ. Thus, we estimated a 646 fg IL4/cell in SPION-IL4/30, and a 1940 fg IL4/cell in both SPION-IL4/100 and Exo IL4 conditions. Hence, SPION-IL4/100 corresponded to the highest SPION concentration to be administered to cells in the present study, with an equivalent number of IL4 molecules to Exo IL4, namely 1940 fg IL4/cell.

**Figure 3:**
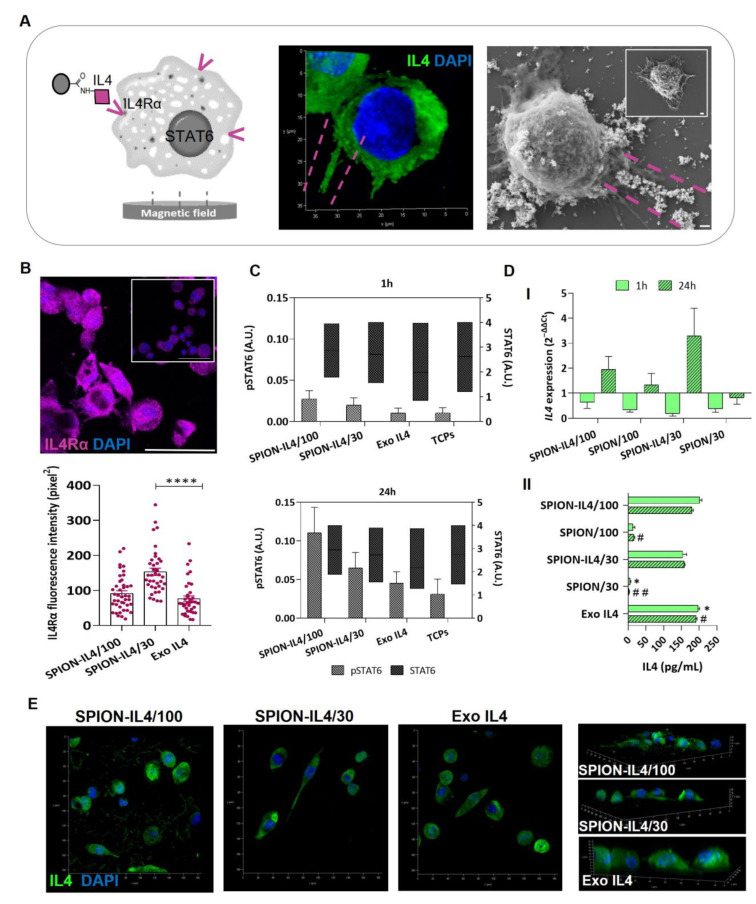
SPION-IL4 interplay in the IL4/STAT6 pathway: from IL4Rα interaction to IL4 production. (**A**) Representative images from confocal microscopy (**left**) and SEM (**right**) of THP1-derived macrophages treated with SPION-IL4/100 for 1 h. The inset in the SEM image represents the negative control (TCPs). Scale bars = 35 µm and 1 µm, respectively. The pink dotted line represents SPION-IL4/100 aligned along the EMF; (**B**) Representative images of IL4Rα (magenta) counterstained with DAPI (blue) 24 h after treatment with SPION-IL4/30. The inset represents Exo IL4 at 24 h. Scale bar = 50 µm; (**C**) STAT6 phosphorylation (pSTAT6) upon treatment with SPION-IL4/30 and SPION-IL4/100, quantified by cell-based ELISA; (**D**) I-gene expression and II-quantification of secreted IL4. In D-I, the expression of IL4 was normalized against GAPDH and to the positive control (Exo IL4) represented by a line at y = 1; (**E**) 3D reconstructed images of IL4 (green) counterstained with DAPI (blue) 24 h after treatment with SPION-IL4. Scale bar = 75 µm. Quantification of the mean fluorescence intensity of IL4Rα and IL4 was performed using ImageJ 1.52o software. Bars represent mean ± SE. Data analysis was performed using the Kruskal–Wallis test followed by Tukey’s HSD test. The symbols * and ^#^ indicate significant differences between groups (*^,#^ *p* < 0.05, ^##^ *p* < 0.01, and **** *p* < 0.0001). All conditions were EMF stimulated for 1 h or 24 h using a magnefect nano device (350 mT/well).

Unidirectional formation of highly oriented rods of SPION-IL4 was observed in the SEM images (Figure 3A), suggesting a highly oriented distribution of SPION-IL4 on cell surfaces in response to EMF application.

To investigate the SPION-IL4 formulation, interactions with IL4 receptor cells were immunostained with an antibody against IL4 receptor alpha (IL4Rα) (Figure 3B). SPION-IL4/100 and Exo IL4 (carrier-free) displayed similar fluorescence intensity values; however, a significant increase was detected in SPION-IL4/30 (*p* < 0.0001). When SPION-IL4 or soluble IL4 binds to the IL4 receptor, the receptor is no longer available for immunostaining. Thus, the signal detected was related to IL4 receptors that were not previously occupied by IL4. Since SPION-IL4/30 is related to a lower concentration of both SPION and IL4 in comparison to SPION-IL4/100 and Exo IL4, more free receptors are available, and thus the signal intensity is higher. Despite the successful delivery of IL4 via SPION-IL4 to the IL4 cognate receptor, the concentrations of SPION-IL4 used did not saturate all the IL4-receptors available in the cells.

Activation of STAT6 is a key signaling in macrophage function, required for the alternative activation of macrophages (M2φ) [27], and indispensable for IL4-mediated activation of target gene transcription as TNFα and interleukin-8 (IL8) [28,29]. Thus, we questioned whether IL4 presentation via EMF-assisted SPION-IL4 could lead to the phosphorylation of STAT6 (pSTAT6) and to M2φ priming via the IL4/STAT6 pathway. Our results showed that the levels of pSTAT6 trended higher in THP1 cells 24 h after SPION-IL4 treatment than in cells supplemented with IL4 only (Exo IL4) and cells cultured on tissue culture polystyrene (TCPs) (Figure 3C). This effect seems to be dependent on SPION-IL4 concentration, and STAT6 responds more effectively to SPION-IL4 than to Exo IL4. To determine whether the THP1 functional fate would also depend on SPION-IL4 concentration, gene and protein expression were assessed. The IL4 followed an incremental expression 24 h after treatment with SPION-IL4, with an almost 2-fold increase in SPION-IL4/30 over SPION-IL4/100 (Figure 3D-I). Intriguingly, IL4-free SPION/100 seemed to have an inherent effect over IL4 expression, which is supported by the fact that SPION treatment in macrophage models altered their M2 activation profiles [30]. In contrast, IL4-free SPION, independently of the iron concentration, did not seem to affect IL4 secretion (*p* < 0.05 and *p* < 0.01) (Figure 3D-II). These data confirm that the functional outcomes of SPION-IL4 relate to IL4 functionalization and are not promoted by the presence of IL4-free SPION. The secretion levels of IL4 when cells were treated with SPION-IL4 were similar to those of Exo IL4 (*p* > 0.05) independently of the iron concentration used (Figure 3D-II). Concordantly, confocal microscopy images showed a similar distribution of intracellular/pericellular IL4 between the SPION-IL4 and Exo IL4 conditions (Figure 3E).

Overall, SPION-IL4 was more effective in the transport and delivery of IL4 participating in the activation of the IL4/STAT6 pathway. The production of IL4 at the gene and post-transcript levels was more efficient when magnetic nano-vehicles were applied, especially with a lower concentration of SPION-IL4 (30 µg/mL), suggesting that higher concentrations of IL4, either provided by soluble IL4 (Exo IL4) or in SPION-IL4 nanocarriers, may lead to suboptimal levels of IL4 mediated signals in THP1-derived macrophages.

### 2.4. Profiling Immune-Modulatory Genes and Inflammatory Mediators upon Treatment with SPION-IL4

**Figure 4:**
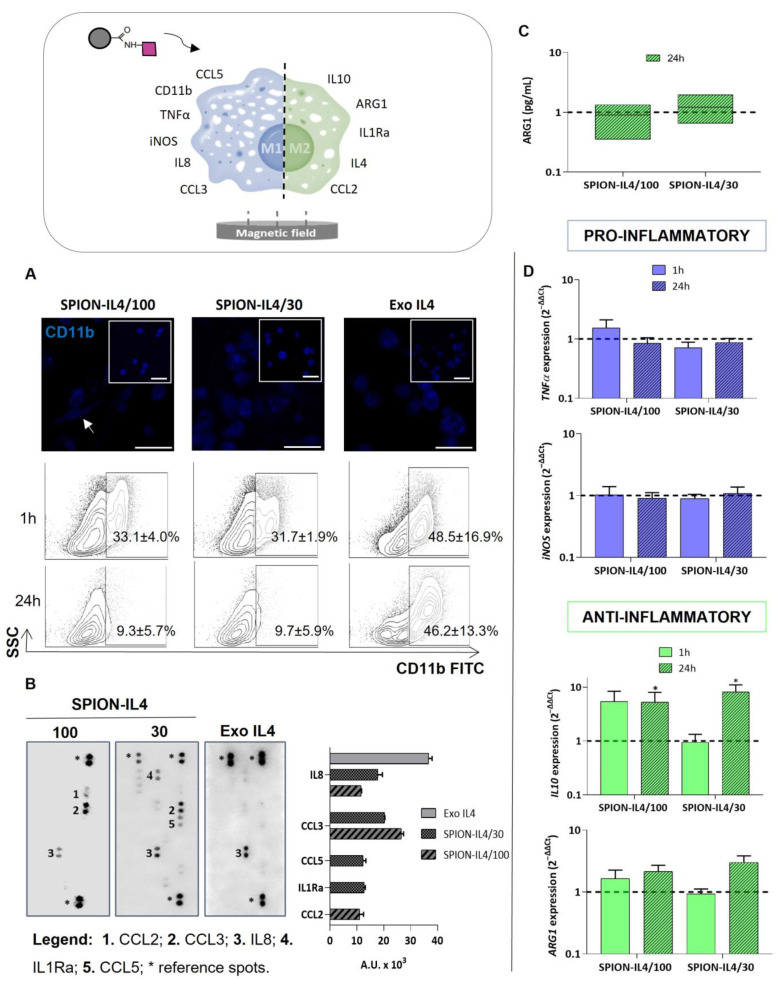
Immune mediators’ profile of THP1-derived macrophages after treatment with SPION-IL4. (**A**) Confocal microscopy images for immunodetection of CD11b (blue) in THP1 cells 24 h after treatment with SPION-IL4/30 and SPION-IL4/100. Insets correspond to the time-point 1 h. Scale bar = 50 µm. The flow cytometry plots indicate the percentage of CD11b^+^ FITC cells; (**B**) Differential expression of cytokines/chemokines associated with inflammation screened by a Proteome Profiler Human Cytokine Array; (**C**) ARG1 quantification in culture medium 24 h after SPION-IL4 treatment; (**D**) Gene expression analysis of pro-(TNFα and iNOS) and anti-(IL10 and ARG1) inflammatory markers associated with macrophage phenotypes. The expression of target genes was normalized against GAPDH and the control (Exo IL4). * indicates significant differences between groups (* *p* < 0.05 for 24 h). Bars represent mean ± SE. Data analysis was performed using the Kruskal–Wallis test, followed by Tukey’s HSD test. All conditions were EMF stimulated for 1 h or 24 h using a magnefect nano device (350 mT/well).

According to the literature, integrin αM (CD11b) has been implicated in the activation of pro-inflammatory transcription factors, such as nuclear factor kappa B (NFKB) [31], as well as in the promotion of pro-inflammatory responses of monocytes and dendritic cells through direct interactions of lipopolysaccharide (LPS) with integrin beta2 extracellular domains [32,33]. The immunodetection of CD11b was evaluated on THP1-derived macrophages both by confocal microscopy and flow cytometry. Initially, the expression of CD11b was similar under all conditions investigated (Figure 4A). However, a clear shift in the number of CD11b^+^ cells was observed after 24 h in SPION-IL4 conditions. The decreased availability of the CD11b receptor on cells treated with SPION-IL4 was accompanied by an elongated morphology (white arrow), which has been associated with a M2φ phenotype [34].

The cocktail of cytokines/chemokines released by macrophages is indispensable for characterizing their polarized states. The cytokine/chemokine profile of SPION-IL4 treated cells was reported for pro- and anti-inflammatory mediators (Figure 4B). IL8 is involved in pro-inflammatory stimulation by activating the classical mitogen-activated protein kinase (MAPK) signaling cascade [35]. The detection of IL8 was shared by all the conditions studied with higher expression in the carrier-free condition (Exo IL4). The common presence may be due to IL8 spontaneous production by cultured primary monocytes and macrophages and differentiated THP1 cells [36]. Nevertheless, IL8 seemed to be modulated more effectively in the presence of SPION-IL4. Intriguingly, CCL3 (C-C Motif Chemokine Ligand-3), a protein secreted by activated macrophages to attract other pro-inflammatory cells and recruit macrophages themselves to sites of inflammation [37], was shared only by SPION-IL4-treated conditions.

The concentration of SPION-IL4 seemed to have a particular effect on the secreted chemokines CCL5 (C-C Motif Chemokine Ligand-5) and CCL2 (C-C Motif Chemokine Ligand-2). The production of CCL5, whose signaling contributes to M1φ polarization and can be inhibited by IL4 [38], was detected in SPION-IL4/30 but not in SPION-IL4/100. It seems that different IL4 amounts, indirectly provided by different SPION-IL4 concentrations, have the potential to stimulate the synthesis of distinct inflammatory proteins. Following this trend, CCL2 is linked to M2φ stimulation [39] and was found only in the SPION-IL4/100 condition. Indeed, it has been reported that virus-induced STAT6 activation leads to the activation of chemokines such as CCL2 [40], which may explain the increased CCL2 in the SPION-IL4/100 condition, in which the highest amounts of pSTAT6 were also found. The production of IL8, CCL3, and CCL5 suggests that the stimulation of macrophage polarization via IL4 does not completely disrupt the synthesis of M1 markers. The co-expression of some M1 and M2 factors may be related to macrophage functional plasticity, and consequently, to their sensing and finely oriented responses to immediate situations. Together with CCL3 and CCL5, IL1Ra (interleukin 1 receptor antagonist) was expressed in the SPION-IL4/30 condition. IL1Ra is abundantly produced by monocytes (0.9–3.0 ng/mL) and by macrophages (9–150 ng/mL) [41] and is a natural antagonist of the interleukin 1 beta (IL1β) signaling pathway [42]. Thus, IL1Ra production via IL4 may be an indirect means of controlling or antagonize the IL1β-initiated inflammatory response, contributing to M2φ functions. In summary, SPION-IL4 conditions favor the production of immunomodulatory molecules with impact during the inflammatory response, particularly of the STAT6-associated gene CCL2 expressed in SPION-IL4/100 treated Mφ, supporting the participation of the SPION-IL4 in the stimulation of the IL4/STAT6 pathway.

Since M2φ polarization is associated with decreased inducible nitric oxide synthase (iNOS) expression and increased ARG1 levels [43], the ratio of ARG1 to iNOS can be an indication of M2φ polarization. There was a tendency for ARG1 to increase in THP1 cells treated with SPION-IL4 at the protein (Figure 4C) and gene (Figure 4D) levels, while iNOS levels remained at baseline values. Similar to iNOS, TNFα levels did not increase in comparison to Exo IL4. The increased expression of IL10 in cells treated with SPION-IL4 (in comparison to the Exo IL4 control, *p* < 0.05) also supports an M2φ shift.

In this study, we investigated the promising role of magnetic nanoparticle-mediated delivery of IL4 aiming at M2φ polarization. SPION-IL4 contributed to IL4-mediated actions and to the expression of STAT6 responsive molecules such as CCL2 and ARG1. In particular, ARG1 together with STAT6 has been described to modulate Mφ phenotypes and to accelerate inflammation resolution [44]. Studies on Mφ-targeted strategies resourcing to SPION are scarce and focus on metabolic pathways associated with cell death or phagocytosis, often resulting in M1 classical activation. SPION are quite small (~309 nm) and do not undergo phagocytosis mechanisms that are typically induced in particles with dimensions over 0.5 µm [45]. Although some individual SPION-IL4 can be internalized without binding to IL4R, these do not compromise the M2-oriented responses of SPION-IL4 treated Mφ, nor the M1-associated markers in THP1 responses shared by all studied conditions. On the contrary, IL4 presentation to THP1 via SPION-IL4 results in increased amounts of M2 associated mediators, even through the delivery of lower dosages of IL4, in comparison to those from the IL4 supplemented to the medium. Unlike soluble IL4 that will rapidly dilute from the administration site, eventually leading to reduced action and adverse side effects, the intrinsic magnetic compliance of SPION-IL4 systems enables their local retention, effective targeting, and guidance of cell fate at a functional level.

## 3. Materials and Methods

### 3.1. Superparamagnetic Iron Oxide Nanoparticles (SPION) Functionalization with IL4

SPION composed of a magnetite core (Fe_3_O_4_) and a dextran -COOH shell were purchased from micromod GmbH (Rostock, Germany, 09-02-252). SPION were functionalized with IL4 (SPION-IL4) by carbodiimide coupling chemistry using 1-ethyl-3-(3-dimethylaminopropyl)-carbodiimide hydrochloride (EDC) (Sigma-Aldrich, Saint Louis, MO, USA, 03450) and N-hydroxysuccinimide (NHS) (Sigma-Aldrich, Saint Louis, MO, USA, 130672). All production steps were performed at room temperature (RT) unless stated otherwise. SPION were mixed with EDC and NHS in 0.5 M MES buffer (Sigma-Aldrich, Saint Louis, MO, USA, 69890) (pH 6.3) for 45 min. Then, activated SPION were washed by magnetic separation with 0.01 M phosphate buffered saline (PBS) (Sigma-Aldrich, Saint Louis, MO, USA, P4417) (pH = 7.4), and mixed with 10 µg of Recombinant Human IL4 (Peprotech, Cranbury, NJ, USA, 200-04) for 3 h. Following a washing step, 25 mM glycine (Sigma-Aldrich, Saint Louis, MO, USA, G8898) in 0.01 M PBS was added, and the mixture was continuously stirred for 30 min. SPION-IL4 was washed three times and resuspended in 1 mL of ultra-pure water. SPION-IL4 was further purified by dialysis (Sigma-Aldrich, Saint Louis, MO, USA, PURX25005) against water for 24 h and stored at 4 °C.

### 3.2. Characterization of SPION-IL4

#### 3.2.1. Physico-Chemical Characterization of SPION-IL4

Fourier transform infrared (FTIR) spectra were recorded with an IRPrestige 21 spectrometer (Shimadzu) using the attenuated total reflectance (ATR) mode over the range of 4000–600 cm*^−^*^1^ with a resolution of 4 cm*^−^*^1^. Analyses were performed on the suspensions collected before (SPION) and after functionalization (SPION-IL4). An ultrapure water background was used before the experiments. SPION-IL4 morphology were analyzed using scanning transmission electron microscopy (STEM) (Auriga Compact, Zeiss, Jena, Germany). The elemental composition of SPION-IL4 was determined using Energy Dispersive X-ray spectroscopy (EDS) (INCAx-Act, PentaFET Precision, Oxford Instruments, High Wycombe, UK). The hydrodynamic diameter and zeta potential of SPION-IL4 were determined using a Malvern NanoZS (Malvern Instruments, Malvern, UK).

#### 3.2.2. Functionalization Efficiency

The functionalization efficiency of IL4 for SPION was evaluated using western blotting. Samples: SPION, SPION-IL4, supernatant from the IL4 incubation solution with SPION (S1), and supernatant from the first washing step with PBS (S2) were analyzed, as well as standard IL4 solutions (0 to 25 µg/mL). A mixture of Laemmli buffer (BioRad, Hercules, CA, USA, 1610737) and the samples (1:1) was incubated at 95 °C for 5 min before running on a 12.5% separation gel and 4% stacking gel (SDS gel preparation kit, Sigma-Aldrich, Saint Louis, MO, USA, 08091). The PageRuler Plus Prestained Protein Ladder (10–250 kDa, Thermo Scientific, Vilnius, Lithuania, 26620) was used as a molecular weight marker. The gel was stained with Coomassie Brilliant Blue (Biorad, Hercules, CA, USA, 1610436) for protein visualization. The gel was transferred to a nitrocellulose membrane (Sigma-Aldrich, Saint Louis, MO, USA, GE10600002) using a Pierce Power System (25 V, 1 mA, 1 h, Thermo Scientific, Vilnius, Lithuania). The membranes were incubated for 1 h with a blocking solution, followed by overnight incubation with recombinant anti-IL4 (1:1000, Abcam, Cambridge, UK, ab62351) at 4 °C. Membranes were washed in Tris-Buffered Saline containing 0.1% Tween^®^20 detergent (TBST, Sigma-Aldrich, Saint Louis, MO, USA, P1379) and incubated with Anti-Rabbit IgG secondary antibody (1:1000, Sigma-Aldrich, Saint Louis, MO, USA, A9919) at RT for 1 h. After washing in TBST, the signal was developed using an AP Conjugate Substrate Kit (Biorad, Hercules, CA, USA, 1706432) according to the manufacturer’s instructions. For the western blotting assay, the supernatant of the SPION-IL4 solution (S) after brief centrifugation was also included as a control for IL4 binding to SPION. Membrane images were acquired using a digital scanner (Epson Perfection V550 Photo, Jawa Barat, Indonesia) in conjunction with Epson Scan software ver. 3.9.2.1US. IL4 bands were further quantified using ImageJ 1.50i (NIH, Wayne Rasband, Kensington, MD, USA) software.

### 3.3. Macrophage Assays

Human monocytes (THP1, ATCC (American Type Culture Collection: TIB-202)) were cultured in Roswell Park Memorial Institute medium (RPMI) (Sigma-Aldrich, Saint Louis, MO, USA, R7755) supplemented with 1% L-Glutamine (Thermo Scientific, Bleiswijk, The Netherlands, 25030) in a humidified 5% CO_2_ atmosphere. THP1-derived macrophages were differentiated with 100 nM phorbol 12-myristate 13-acetate (PMA; Sigma-Aldrich, Saint Louis, MO, USA, P8139) for 24 h, followed by 24 h incubation with a PMA-free medium. Adherent THP1-derived macrophages were cultured and expanded in RPMI medium. Two time-points (1 h or 24 h) and up to four conditions were investigated: THP1 cells cultured with (i) SPION-IL4 at 30 µg/mL or (ii) SPION-IL4 at 100 µg/mL of iron (SPION-IL4/30 or SPION-IL4/100, respectively) and non-functionalized SPION at (iii) 30 µg/mL or (iv) 100 µg/mL of iron (SPION/30 or SPION/100, respectively). Conditions (iii) and (iv) act as experimental controls for IL4 functionalization. Magnetically stimulated THP1 cells exogenously supplemented with soluble IL4 were used as a positive control (hereafter referred to as Exo IL4) whereas cells cultured on tissue culture polystyrene (TCPs) were used as a negative control. The concentration of IL4 per cell used in the SPION-IL4/100 and Exo IL4 conditions was the same. EMF was provided by a magnefect nano device (nanoTherics Ltd., Warrington, UK) (350 mT/well) under all conditions.

### 3.4. Cytotoxicity Assessment

To determine the effect of SPION-IL4 on cells, THP1-derived macrophages were seeded at 1 *×* 10^5^ cells per well (24 wells, Corning, AZ, USA, 353047), followed by incubation with SPION-IL4 at 37 °C under magnetic stimulation. For LDH experiments, cells were seeded at 1 × 10^4^ cells per well (96 wells, Biotecnomica, Herlev, Denmark, TPP92096). Live/dead cell double staining: Cells were washed with PBS and incubated with 2 µM calcein AM in PBS (Thermo Scientific, Eugene, OR, USA, C3099) and 4 µM propidium iodide (PI) (Thermo Scientific, Eugene, OR, USA, P1304 MP) in the dark for 30 min at 37 °C. Cells were rinsed in PBS, and the fluorescence signal was acquired using a transmitted and reflected light microscope with Apotome 2 (Axio Imager Z1 m, Zeiss). Cell viability was assessed using both 3-(4,5-dimethylthiazol-2-yl)-5-(3-carboxymethoxyphenyl)-2-(4-sulfophenyl)-2H-tetrazolium (MTS) and LDH assays. MTS: THP1 cells were incubated in MTS solution (1:5, Promega, Madison, WI, USA, G3581) for 3 h at 37 °C in a 5% CO_2_ atmosphere and protected from light. The supernatant was transferred to a new 96-well plate and the optical absorbance was measured at 490 nm (SynergyTM HT, BIO-TEK Instruments, Winooski, VN, USA). LDH: At the respective time-points, cytotoxicity was measured in each sample medium using a CyQuant LDH Cytotoxicity Assay kit (Thermo Scientific, Eugene, OR, USA, C20301), following the manufacturer’s instructions. LDH activity was measured at 490 nm/680 nm excitation/emission wavelengths using a microplate reader (BioTek Synergy HT, Winooski, VT, USA).

### 3.5. Characterization of Macrophages upon Contact with SPION-IL4

#### 3.5.1. RNA Isolation and Gene Expression Analysis

Total RNA was extracted using TRI reagent^®^ RNA Isolation Reagent (Sigma-Aldrich, Saint Louis, MO, USA, T9424) following the manufacturer’s instructions. RNA was quantified using a Nanodrop^®^ ND-1000 spectrophotometer (Thermo Scientific, Eugene, OR, USA) at 260/280 nm. First-strand complementary DNA was synthesized from 1 µg of RNA from each sample (qScriptTM cDNA Synthesis Kit, Quanta Biosciences, Gaithersburg, MD, USA) in a 20 µL reaction using a Mastercycler^®^ ep realplex gradient S machine (Eppendorf). Transcripts were quantified by quantitative polymerase chain reaction (qPCR) using the PerfeCTA SYBR Green FastMix kit (Quanta Biosciences, Gaithersburg, MD, USA) following the manufacturer’s protocol in a Real-Time Mastercycler ep realplex thermocycler (Eppendorf). Primer sequences (Supplementary Materials, Table S1) were designed using Primer 3 software. The 2−∆∆Ct method was used to evaluate the relative expression level for each target gene. An untreated condition (THP1 without magnetic stimulation) was used to determine the relative expression, and the transcript expression of target genes (IL4, TNFα, iNOS, IL10, and ARG1) was normalized to the expression of the endogenous housekeeping gene glyceraldehyde-3-phosphate dehydrogenase (GAPDH). Normalization of the gene expression values was also performed against Exo IL4 (for 1 h or 24 h time-point, respectively), which are represented in the graphs in the form of a dotted horizontal line at y = 1.

#### 3.5.2. Determining the Phosphorylation of STAT6

The relative amount of STAT6 (pTyr641) phosphorylation (pSTAT6) used to screen the effects of SPION-IL4 in THP1-derived macrophages was assessed using a cell-based phospho-STAT6 (pTyr641) ELISA kit (Sigma-Aldrich, Saint Louis, MO, USA, RAB0452) following the manufacturer’s instructions.

#### 3.5.3. Immunodetection of IL4Rα, IL4, and CD11b

IL4 Receptor Alpha (IL4Rα) is the alpha chain of the IL4 receptor and is involved in the regulation of alternative activation of macrophages and, consequently, IL4 secretion levels, whereas CD11b is an integrin family member involved in the regulation of leukocyte adhesion and migration to mediate the inflammatory response. To detect these molecules, THP1-derived macrophages were fixed in 4% neutral buffered formalin (Thermo Scientific, Eugene, OR, USA, 5701) overnight and stored in PBS at 4 °C until use. Subsequently, the cells were incubated with 0.025% Triton-X100 (VWR, Thermo Scientific, Eugene, OR, USA, A16046.AE) in PBS, and blocking was performed using 2.5% normal horse serum (Vector Laboratories, Newark, OH, USA, S-2012). Cells were incubated overnight at 4 °C with anti-IL4Rα (1:200, Thermo Scientific, Eugene, OR, USA, PA5-103142), anti-IL4 (1:500, Abcam, Cambridge, UK, ab62351), or anti-CD11b (1:100, Abcam, Cambridge, UK, ab52478) diluted in 0.1% bovine serum albumin diluted (BSA, Sigma-Aldrich, Saint Louis, MO, USA, A2153), followed by incubation for 1 h at RT with the secondary antibody (1:500, Anti-Rabbit IgG Alexa Fluor 488, Thermo Scientific, Eugene, OR, USA, A21206). The samples were washed three times with PBS before staining with DAPI (5 µg/µL, Sigma-Aldrich, Saint Louis, MO, USA, D9564) for 10 min. Immune detection was then assessed by confocal laser scanning microscopy, and 3D reconstruction images obtained from the z-stacks were obtained using TCS SP8 software (Leica, Mannheim, Germany). IL4Rα and IL4 fluorescence intensities were quantified using ImageJ 1.50i software (NIH, Wayne Rasband, Bethesda, MD, USA).

In the case of CD11b, the number of cells positive for CD11b was determined by flow cytometry. The cells were permeabilized and incubated with anti-CD11b (Abcam, Cambridge, UK, ab52478) for 20 min, followed by incubation with the secondary antibody (Anti-Rabbit IgG Alexa Fluor 488) for 30 min in the dark. Cells were then washed with PBS and resuspended in acquisition buffer (1% formaldehyde in PBS) before acquisition on a FACSAria III sorter equipped with blue and red lasers (BD Biosciences, Erembodegem-Aalst, Belgium). The cells were identified using forward and side scattering. A minimum of 5000 cells were acquired and analyzed using the FACS Diva software version 7. Unstained cells were used to establish cell autofluorescence, and cells incubated only with secondary antibodies were used as controls. The population of cells positive for CD11b was expressed as a percentage.

#### 3.5.4. SPION-IL4/Cells Surface Interaction Analysis by SEM

Scanning electron microscopy (SEM) was used to visualize the physical distribution of SPION-IL4 in the cells. THP1-derived macrophages were fixed in 4% neutral buffered formalin (Thermo Scientific, Eugene, OR, USA, 5701) overnight and dehydrated using ascending concentrations of ethanol (from 30 to 100%), followed by a 5 min immersion in hexamethyldisiloxane (HMSO, Sigma-Aldrich, Saint Louis, MO, USA). The samples were then air-dried overnight and sputter-coated (30 s at 20 mA, Cressington, UK, C5219, Model 108 A) with gold. Images were acquired using scanning electron microscopy (SEM; JEOL, Japan).

#### 3.5.5. Cytokine Production Assays

For the cytokine arrays, 3 *×* 10^5^ THP1-derived macrophages per well were cultured in 24 wells plates (Corning, Arizona, USA, 353047) before supplementing with SPION-IL4 and IL4. After 24 h, the supernatants were collected and analyzed using a Proteome Profiler™ Human Cytokine Array Kit (R&D Systems^®^, Minneapolis, MN, USA, ARY005B) following the manufacturer’s instructions. The spot intensity was detected using the Odyssey Fc Imaging System 2800 (LI-COR^®^) and quantified using ImageJ 1.52o (NIH, Wayne Rasband, Bethesda, MD, USA) software. Supernatants were also analyzed for the quantification of secreted cytokines using commercially available enzyme immunoassay kits for ARG1 (Arginase-1 Human ELISA Kit, Thermo Scientific, Eugene, OR, USA, BMS2216) and IL4 (Human IL4 ELISA Kit, Sigma-Aldrich, Saint Louis, MO, USA, RAB0298) following the manufacturer’s instructions.

### 3.6. Statistical Analysis

Results are expressed as the mean ± standard error of the mean (SE), representative of three independent experiments *(n* = 3), except for flow cytometry analysis of CD11b, which is representative of two independent experiments (*n* = 2), with a minimum of 5000 cells analyzed per experiment. Statistical analysis was performed using SPSS statistical software (version 27.0.1.0). First, a Shapiro–Wilk test was used to ascertain data normality and Levene’s test for homogeneity of variances. Normality and variance homogeneity were rejected, and non-parametric tests were used (Kruskal–Wallis test followed by Tukey’s HSD test). Different degrees of confidence were considered, *p* < 0.05, *p* < 0.01, *p* < 0.0001, and are represented by symbols * and # for *p* < 0.05, **, ## for *p* < 0.01, and **** for *p* < 0.0001.

## 4. Conclusions

This study showed that SPION-IL4 is a suitable IL4 presenter, anticipating the modulation of cell behavior with insights into macrophage-priming strategies mediated by EMF-actuated SPION. SPION-IL4 treated Mφ showed an increased expression of genes such as IL10 and ARG1 and proteins (CCL2 and IL1Ra) typically associated with the M2φ phenotype, with unique advantages over carrier-free administration. Together with the therapeutic effect, the magnetic memory of SPION-IL4 comprises multimodal possibilities, including cell tracking and in vivo identification of IL4-targeted populations.

In view of the presence of macrophages in almost all tissues and organs, SPION in combination with IL4 and/or other cytokines could hold wide applicability as phenotype modulators and, consequently, as magnetically responsive switches of pro- and anti-inflammatory activities. Either in the form of magnetic delivery systems or magnetically responsive elements incorporated into tridimensional matrices, these systems could render immunomodulatory actions foreseeing improved therapeutic solutions for chronic inflammation conditions and tissue regeneration.

## Data Availability

The data presented in this study are available on request from the corresponding author.

## Supplementary Materials

The following supporting information can be downloaded at: https://www.mdpi.com/article/10.3390/ijms232315125/s1, Figure S1: TGA analysis of the non-functionalized SPION for the quantification of inorganic and organic components present in the nanoparticles; Figure S2: SDS-PAGE gel analysis to unravel the binding efficiency of IL4 to SPION; Figure S3: Semi-quantitative calculation of IL4 concentration in SPION-IL4 samples based on ImageJ analysis of western blotting bands; Table S1: Primers used for real time quantitative RT-PCR analysis.
