# Supplementary material for: Controlling Macrophage Polarization to Modulate Inflammatory Cues Using Immune-Switch Nanoparticles
Source: Int J Mol Sci. 2022 Dec 1;23(23):15125. doi: 10.3390/ijms232315125 (PMC9739781; doi:10.3390/ijms232315125)
Supplement: Supplementary file 1 [file ijms-23-15125-s001.zip › ijms-2067268-supplementary/Supporting Information_25112022.pdf]

Supporting Information

Controlling macrophage polarization to modulate inflammatory cues using immune-switch nanoparticles

Ana F. Almeida, Margarida S. Miranda, Adriana Vinhas, Ana I. Gonçalves, Manuela E. Gomes, Márcia T. Rodrigues**

Figure S1. TGA analysis of the non-functionalized SPION for the quantification of inorganic and organic components present in the nanoparticles.

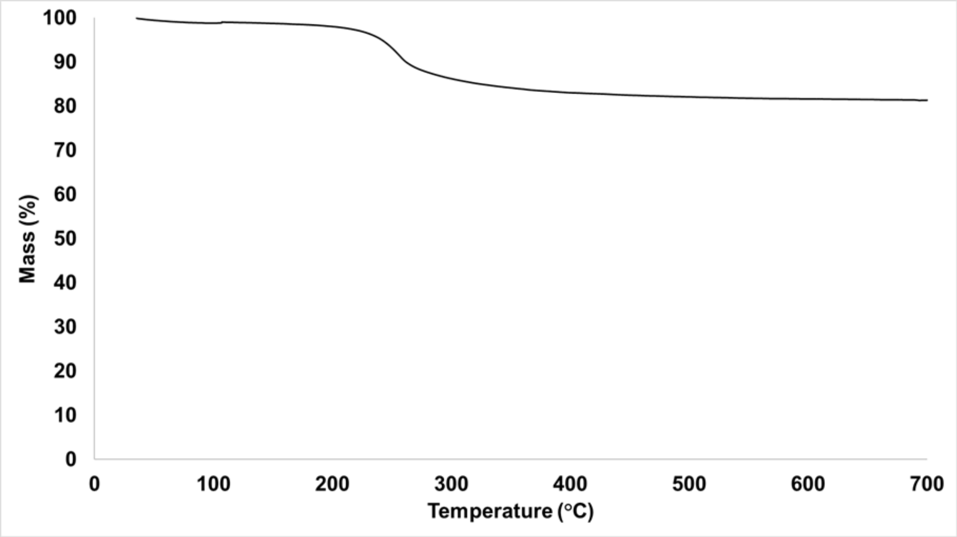

The inorganic and organic components of the non-functionalized SPION was evaluated by thermal gravimetric analysis (TGA) with a simultaneous thermal analyzer (model STA7200, Hitachi). Dry SPION (1-2 mg) were placed in a platinum crucible, using an empty crucible as reference. The temperature of the furnace was raised to 105°C at a rate of 10°C min^{-1} and then held at this temperature for 15min, under a nitrogen atmosphere, to remove the still adsorbed water. The temperature was then increased to 700°C at 20°C min^{-1} under an oxygen atmosphere and held at this temperature for 10min in order to completely burn the organic component of the SPION. Figure S1 shows a TGA experiment of the dried non-functionalized SPION where the mass change in percentage was recorded as function of the temperature.

Supporting Information

Controlling macrophage polarization to modulate inflammatory cues using immune-switch nanoparticles

Ana F. Almeida, Margarida S. Miranda, Adriana Vinhas, Ana I. Gonçalves, Manuela E. Gomes, Márcia T. Rodrigues**

Figure S2. *SDS-PAGE gel analysis to unravel the binding efficiency of IL4 to SPION.*

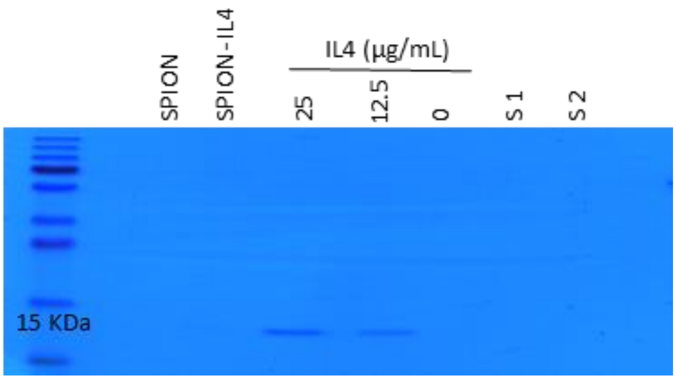

SDS-PAGE analysis with Coomassie blue staining. IL4 standard solutions (0 to 25 µg/mL) were used for the detection of soluble IL4. S1 and S2 refer to supernatants collected from IL4 incubation solution with SPION (S1), and after the first washing step with PBS (S2).

Supporting Information

Controlling macrophage polarization to modulate inflammatory cues using immune-switch nanoparticles

Ana F. Almeida, Margarida S. Miranda, Adriana Vinhas, Ana I. Gonçalves, Manuela E. Gomes, Márcia T. Rodrigues**

Figure S3. Semi-quantitative calculation of IL4 concentration in SPION-IL4 samples based on ImageJ analysis of western blotting bands.

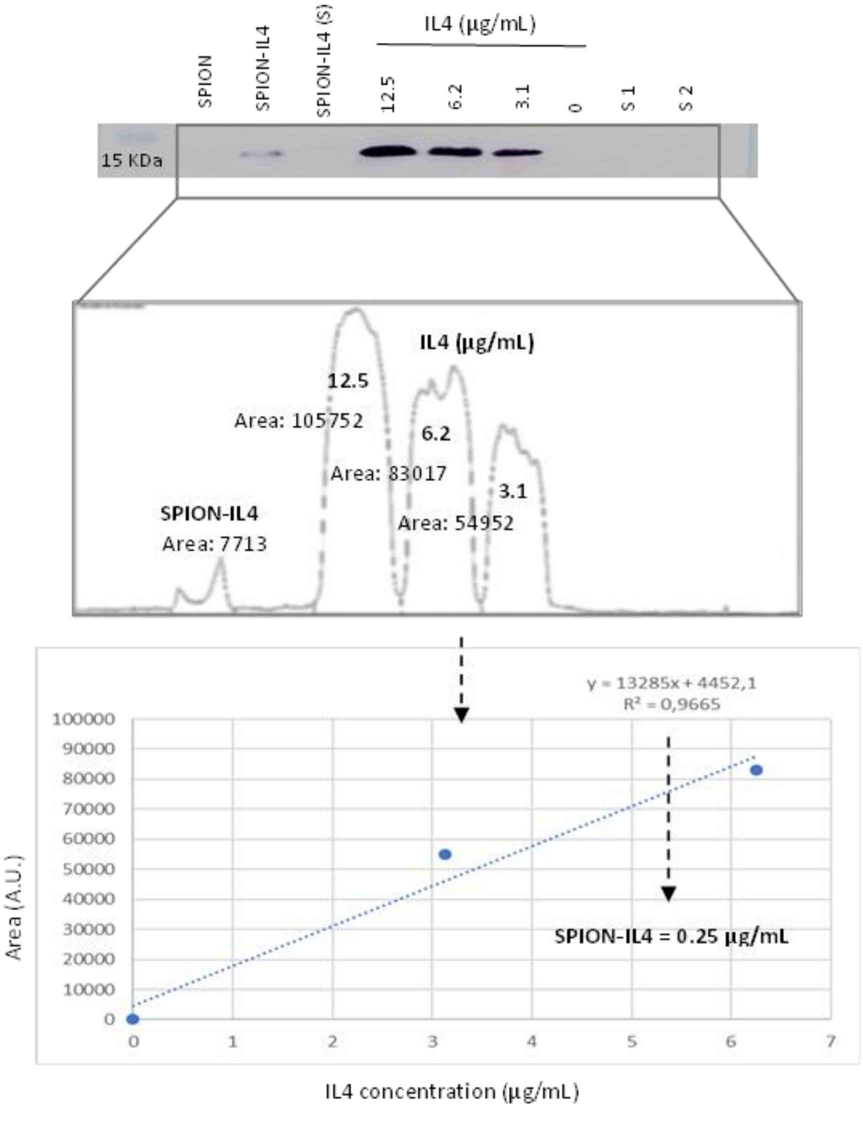

Supporting Information

Controlling macrophage polarization to modulate inflammatory cues using immune-switch nanoparticles

Ana F. Almeida, Margarida S. Miranda, Adriana Vinhas, Ana I. Gonçalves, Manuela E. Gomes, Márcia T. Rodrigues**

Table S1. Primers used for real time quantitative RT-PCR analysis.

	Primer sequence	Accession number
**Human Glyceraldehyde 3-phosphate dehydrogenase (*GAPDH*)**	F: TGTACCACCAACTGCTTAGC<br>R: GGCATGGACTGTGGTCATGAG	NM_002046.4
**Human interleukin-4 (*IL4*)**	F: GCACCGAGTTGACCGTAACA<br>R: AGGAATTCAAGCCCGCCAG	NM_000589.3
**Human nitric oxide synthase 2 (*iNOS*)**	F: GGACATCGCGTGGGTGAA<br>R: TTTATCGCTCGGAGCCTGC	NM_000625.4
**Human tumour necrosis factor (*TNFα*)**	F: ATGTTGTAGCAAACCCTCAAGC<br>R: TGATGGCAGAGAGGAGGTTG	NM_000594.3
**Human interleukin-10 (*IL10*)**	F: AAGACCCAGACATCAAGGCG<br>R: AATCGATGACAGCGCCGTAG	NM_000572.2
**Human arginase-1 (*ARG1*)**	F: GGAAAACCAAGTGGGAGCAT<br>R: TGTGGTTGTCAGTGGAGTGT	NM_000045.4
